# HTS and hit finding in academia – from chemical genomics to drug discovery

**DOI:** 10.1016/j.drudis.2009.09.004

**Published:** 2009-12

**Authors:** Julie A. Frearson, Iain T. Collie

**Affiliations:** Drug Discovery Unit, Sir James Black Centre, College of Life Sciences, University of Dundee, Dow Street, Dundee DD1 5EH, UK

## Abstract

The liaison between academia and the pharmaceutical industry was originally served primarily through the scientific literature and limited, specific industry–academia partnerships. Some of these partnerships have resulted in drugs on the market, such as Vorinostat (Memorial Sloan-Kettering Cancer Centre and Merck) and Tenofovir (University of Leuven; Institute of Organic Chemistry and Biochemistry, Czech Republic; and GlaxoSmithKline), but the timescales from concept to clinic have, in most cases, taken many decades. We now find ourselves in a world in which the edges between these sectors are more blurred and the establishment and acceptance of high-throughput screening alongside the wider concept of ‘hit discovery’ in academia provides one of the key platforms required to enable this sector to contribute directly to addressing unmet medical need.

## Introduction

The days of a clear distinction between academia's and industry's roles in developing new therapies are long gone and, in the past ten years, a small revolution has taken place within institutions previously recognized as centres of international excellence in fundamental research. Many such institutions now have translational research-based groups and platforms developed to take basic research and apply it towards clinical applications. Academia has for some time recognized the role of identification of chemical matter in the endeavour of addressing specific biological questions, and the stochastic nature of screening for these molecules is now widely accepted as a legitimate discipline in this sector. There are many academic contextualized screening centres across the world, each with different specializations and remits and, collectively, they represent a vast transfer of knowledge and technology bases from industry into academia. The resulting blend of academic endeavour and innovation with industrial purpose and discipline provides fertile grounds for translational research. It is anticipated that the drive from funding bodies towards translational research will sustain these activities as they continue to address chemical genomics and, increasingly, enter the world of drug discovery. While the pharmaceutical sector has to moderate its strategy to cope with challenging business and economic times, the academic drug discovery sector has the potential to develop to a level of maturity and robustness with which it can contribute meaningfully to the development of new mode-of-action therapies against a range of diseases, not least neglected and niche diseases. In this review, we aim to give an overview of the current academic screening sector and provide opinions on areas for future focus and development. We use the terms ‘academia’ and ‘academic’ to encompass both teaching and non-teaching institutes, plus some other not-for-profit centres, and to distinguish them from the traditionally recognized pharmaceutical and biotechnology sectors.

## Stakeholders in academic screening

In addition to the university system itself, there are several other bodies that have important roles in funding, overseeing, translating and guiding the research and screening output in the academic sector. Government-funded bodies, such as the Medical Research Council (MRC) in the UK and the National Institutes for Health (NIH) in the US, have always invested in leading-edge research activities within universities and research institutes, but over the past decade a noticeable focus on drug discovery initiatives and screening facilities has been evident: for example, see the MRC's Translational Research Strategy [1] and the NIH Roadmap Initiative [2]. Charitable and philanthropic funding is also a notable source of support for the translational research sector, including the Wellcome Trust's Seeding Drug Discovery initiative [3], Cancer Research UK's drug discovery operations (the Institute of Cancer Research Centre for Cancer Therapeutics, and Cancer Research Technology) and the Broad Institute. Furthermore, public–private partnerships also use academic screening centres to push forward their drug discovery portfolios; for example, the Drugs for Neglected Diseases Initiative in partnership with the Drug Discovery Unit (DDU) at Dundee [4] and Medicines for Malaria Venture working with the Broad Institute [5].

**Figure 1:**
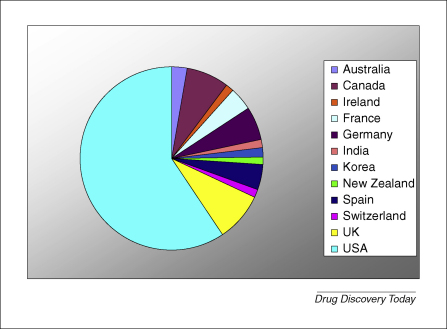
Geographical distribution of the majority of existing academic screening centres (data drawn primarily from the SBS Academic Screening Facilities Directory at http://www.sbsonline.org/).

The Society of Biomolecular Sciences Reference Library [6] currently lists 64 different institutes around the world in its Academic Screening Facility Directory; 59% of these are based in the USA (Fig. 1). Rather than exist in isolation, some of these groups have formed national networks to realize mutual benefits (e.g. the US Molecular Library Screening Centres Network [7], the European ChemBioNet [8] and the UK Drug Discovery Consortium [9]). Box 1 summarizes some representative examples of academic screening facilities found around the world. These groups each differ in their remits, which strongly dictate the nature of the platforms and technologies they have established. One area of the sector is dominated by the need to provide fully flexible platforms to capture the widest possible range of ‘biologies’, develop and release probes into the scientific community and make all screening data open access. The provision to the community of single-point screening data in the absence of exposure to the underlying quality control data and predictable interference of data sets with false positives, however, can undermine the utility of this worthy intention. The NIH Chemical Genomics Centre (NCGC) has addressed this through the development and validation of a quantitative high-throughput screening (HTS) platform that provides concentration–effect data for all compounds tested, resulting in data with sufficient richness to engender confidence in activity and initial structure–activity relationships (SARs) for the browsing scientist [10]. Other centres operate with a similar community-based spirit but directed more towards the endeavour of drug discovery for neglected diseases, in which the outputs are leads and preclinical candidates for suitably positioned partners to develop further. Such organizations, therefore, focus their technologies and platforms on serving the needs of screening in the context of the disease-causing organisms and information sharing within defined project teams and consortia. The DDU at Dundee (Box 1) represents a case in point.

## Primary technologies

Where once academic laboratories were devoid of the automation instrumentation that was increasingly deployed in the pharma and biotech sectors of the industry during the 1990s, many of the academic screening centres (Box 1) are now equally well equipped to conduct medium- and high-throughput screening activities (Table 1). Several changes have encouraged these developments, including the availability of commercial compound library sets, price maturation of the automation instrumentation sector, de-specialization of systems and software, and movement of a skills base from industry to academia. Many of the screening centres located in academia have been able to recruit industry-experienced personnel into their groups and, hence, the establishment and implementation of well-established screening technologies have been facilitated to the point where learning curves are being abridged.

**Figure 2:**
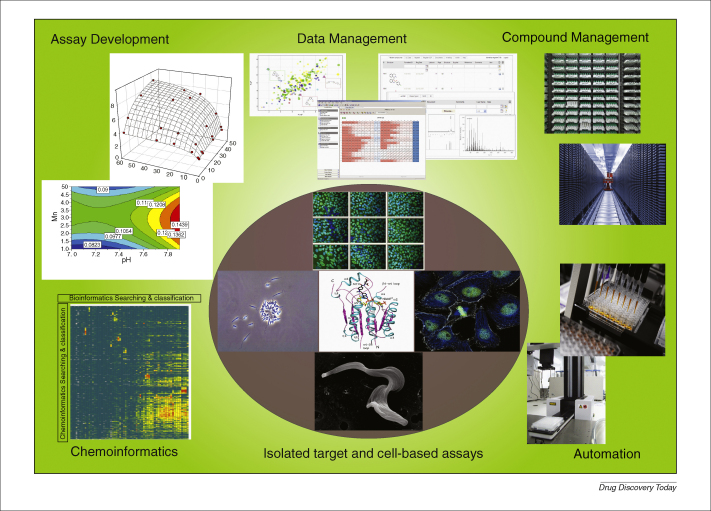
Representation of some key elements underpinning an effective screening facility, including (clockwise, from top left): example of a rectangular design experiment to optimize biochemical assay parameters, as depicted using MODDE V8 software (Umetrics); 2D scatter plot depicted using Vortex software (Dotmatics); plate representation of compound potency depicted using ActivityBase XE software (IDBS); chemical registration view using Register software (Dotmatics); compound storage facilities (REMP); automated liquid handling instrumentation; single-field images of plated cultured embryonic stem cells stained for a pluripotency marker (upper), leishmania promastigotes (left), ribbon diagram of *Trypanosoma brucei* pteridine reductase (centre), *Xenopus* XLK2 kidney cell in mitosis (right), electron micrograph of *T. brucei* (lower); and a bioinformatics array plot of screens (*x*-axis) against compounds (*y*-axis) where compound potency is displayed as a colour gradient from red (most active) to green (inactive).

The discipline of hit discovery is underpinned by a raft of technology requirements including a plethora of assay formats: liquid handling, automation, large data set management and chemoinformatics (Fig. 2). The extent and nature of the infrastructure required by each screening centre is driven by their area of specialism and the size of their screening collections. These typically vary between 50,000 and 300,000, with screens generally being run in 384-well plate formats. It is common to find laboratories adopting a workstation approach with modular ‘islands’ of automation rather than fully integrated robotic installations, with the notable exception of the larger ultra-HTS initiatives engaging in 1536-well plate densities. In particular, the NCGC have developed a 1536-based robotic platform to support their quantitative HTS [11].

The majority of centres have a range of the conventional biochemical assay options that use all of the well-established photo-detection technologies (absorbance, luminescence, fluorescence intensity, time-resolved fluorescence, fluorescence polarization, fluorescence resonance energy transfer and time-resolved fluorescence resonance energy transfer) underpinning both molecular-target- and cell-based screening. These formats are often supported by a range of detectors, from standard multi-modal readers to laser scanning cytometry, ultra-fast charge-coupled-device-based wide field imaging and ultimately confocal high-content screening systems. Some centres support their specialist interests with whole-organism screening in protozoan parasites (DDU, Dundee) and model multicellular organisms, such as zebrafish [12], nematodes and fruit flies [13]. Target-family-based or technology-based specialist centres also exist, including John Hopkins University Ion Channel Centre [14], which specializes in high-throughput label-free technologies, automated patch clamping and atomic absorption spectroscopy, and the University of New Mexico Centre for Molecular Discovery Capabilities [15], where high-throughput flow cytometry is performed in 384-well format.

The importance of high stringency assay development in this sector cannot be overestimated. This subject belies one of the main cultural and operational distinctions between academic and industry-trained scientists. The concept of developing biological assays and systems with sufficient robustness to operate at scale for screening and then with longevity to support chemical probe development or drug discovery is the one that brings distinct challenges when adapting assay concepts from research laboratories. In recognition of this, the NCGC, in collaboration with investigators from Eli Lilly, have compiled an Assay Guidance Manual [16] to encourage good practice among the wider scientific community.

In the past, screening centres have tended to develop in isolation from the associated technologies and capabilities required to make their outputs of the highest value. The most effective groups in the sector recognize the importance of stringent compound management [17,18] and have fully integrated capabilities in chemoinformatics, synthetic organic chemistry and/or medicinal chemistry. Having closely coupled chemistry platforms in these centres is a key component because they play a vital part in informing the design and selection of compound libraries, enabling rapid validation of hit series through resynthesis and early SAR development and enabling the required dissemination of data, be it internally to drug discovery project teams or externally through open-access mechanisms.

## Where do the hits come from?

Lessons learned over several years have now produced the concept of a healthy balance of options for the discovery of chemical matter for biological systems involving high-quality (although not necessarily high-throughput) screening of well-designed, appropriately propertied and enriched small molecule compound libraries, besides the use of alternate approaches, including natural products and small molecular fragments.

### Small molecules

Small-molecule-based high-throughput screening has had a chequered history, even within its ancestral home of industrial drug discovery. Much of the controversy surrounding its effectiveness is born out of other trends in the pharmaceutical sector that happened in parallel; notably, the advent of combinatorial chemistry and the molecular genomic revolution. When all three major events connected – the availability of millions of chemistries, hundreds of new molecular targets and the systems to permit large-scale cross-screening – the outcome was one of considerable investment without the anticipated stream of new chemical entities progressing towards the clinic [19]. Despite this, there are now examples of molecules in the clinic that originated from high-throughput screens, including agents used in the treatment of HIV infection (Maraviroc [20] and Viramune [21]).

**Figure 3:**
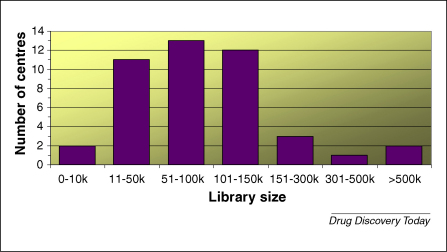
Relative size of compound collections held in 44 of the academic screening facilities (data drawn from the SBS Academic Screening Facilities Directory).

Most academic centres focus on small-molecule screening, and many have several compound collections (Fig. 3) incorporating both diversity-based and gene-family or target-focussed compound sets, applying them strategically to individual screening campaigns. The content, design and scale of the small molecule library in a screening centre needs to be directly related to its intended purpose. There might be more scope for molecular weight distribution in libraries destined for chemical probe discovery, but beyond that, the property restrictions for lead-like drug discovery requirements also apply to chemical biology scenarios [22]. The avoidance of structures with promiscuous inhibitor tendencies, toxicophores, reactive moieties and intractable chemistries are relevant in both contexts. In addition, libraries intended for drug discovery utility need to further adhere to a restricted range of physicochemical properties necessary for both parenteral or oral bioavailability and, perhaps, central nervous system penetration [23,24]. The optimum size of the compound collection required to maximize diversity and to provide sufficient representation to provide early SAR from the screening exercise is a subject of much debate. The desire to screen large sets is born from the need to identify a broad range of hit series, a crucial point in the competitive arena of industrial drug discovery. In academic screening centres, the compound sets need to cover the appropriate biology space; as much chemistry space as possible and – if chemistry resource is limited for the elaboration of the hits and, thereby, validation of the emergent series – a good level of representation around core scaffolds is required to provide information on the most robust series. Academic centre libraries have, in the majority, been collated from scratch and derived from commercial sources, allowing a design element to influence their content [25]. When purchasing from commercial space, it is crucial to apply property filters and cherry pick compounds for inclusion to avoid unnecessary over-representation of scaffolds. The University of Dundee's analysis of >4 million commercially available structures revealed only ∼300,000 that were appropriately propertied for inclusion into a drug discovery library and, after core fragment analysis, it was concluded that these 300,000 structures could be adequately represented by 80,000–100,000 compounds. Example compound selection criteria for some screening centres have been published [26,27]. The NIH Molecular Libraries Initiative [28] is undoubtedly the largest academic compound collection. It currently holds approximately 300,000 compounds that cover specialty sets, natural products, targeted libraries and diversity sets. A full comparative analysis of all libraries in centres described in Box 1 has not been undertaken. However, a small-scale assessment at Dundee (R. Brenk, personal communication, details to be published elsewhere) of libraries from five different groups revealed a large overlap at the core fragment level and minimal overlap at the compound level.

### Natural products

Many precedents have been set regarding the utility of natural products as therapeutics, including lovastatin [29], derived from a fungus, and paclitaxel [30], derived from tree bark. Modern day drug discovery has moved away from this endeavour because of the complexity of identifying, purifying and chemically optimizing the active components. Nevertheless, academia has long been associated with the investigation of these entities, and academic screening, in many respects, owes its origins to this field. Most centres have a natural product ‘aspect’ to their compound collections, and these natural products might often be used when small-molecule probes are not easily identified; some centres specialize in this offering. The Eskitis Institute for Cell and Molecular Therapies at the Griffith University in Brisbane has access to the ‘Nature Bank’ (Queensland Compound Library), a bank of more than 250,000 compounds from plants and marine invertebrates ready for drug trials. The Nature Bank has taken more than ten years to assemble and comprises unique compound samples collected from flora and fauna across Australia, parts of China and the remote tropical forests of Papua New Guinea. The utility of the hit output from these sets is highly dependent upon aligned natural product chemistry and microbiology expertise [31].

### Fragments

Fragment-based hit discovery offers new possibilities for academic screening groups, providing the opportunity to probe large areas of chemical space in small-scale screening campaigns with a technology that is now a proven method of hit identification in industry (for a review, see Ref. [32]). The primary concept behind fragment-based hit discovery is that chemical space can be more efficiently probed by screening collections of small fragments (<250 Da). The initial fragments can then be optimized efficiently, in many cases, to give compounds with potencies superior to those from small-molecule screens and that lie well within Lipinski's rules. The low initial affinities returned by these fragments necessitate an increased repertoire of assay technologies to support standard bioassay. Although visualization of key fragments in X-ray-derived structures is still required to develop fragments into small molecules with good potency, a range of additional biophysical techniques to screen for fragment hits, such as surface plasmon resonance (SPR), isothermal titration calorimetry and thermal shift, has made the area generally more accessible. The development of a fragment approach to hit discovery is, in many ways, highly amenable to the academic drug discovery setting, and such groups can apply this technique to the difficult, high-risk target types that should be characteristic of the sector. In particular, a wealth of opinion is now building to suggest that fragment-based hits are the way forward for targeting protein–protein interactions [33–36].

## Focus for the future

When faced with industry colleagues, they often ask the question of academic screening and drug discovery: ‘what are you doing that is different?’ Overall, we should be engaging in projects and programmes that seek to complement and not overlap with activities in industry; working on neglected disease is a case in point. We might, on the whole, use the same battery of methodology and approaches, but these will be applied to distinctive, early-stage targets and mechanisms for which the tractability or druggability has yet to be proven. These are the projects that offer the real opportunity to provide new mode-of-action therapeutics for genuine unmet need. This sector also has the freedom to develop new drug discovery paradigms, seeking to change the well-trodden paths and methods of drug discovery to open up new target classes, new mechanisms of action, new cell models and, perhaps, one day addressing the ultimate goal of strategic polypharmacology [37]. How can hit discovery and screening itself contribute to this raft of potential innovations? Certainly, the primary focus for development in recent years has been the drive for continued miniaturization by the industry; however, this is likely to diminish because many operations become more individual-project- and focussed-compound-set-orientated [38]. Therefore, a new set of advances in hit discovery are now required, focussing on efforts to identify and validate new targets, support drug discovery against new mechanisms and improve the overall efficiency of hit selection. The academic hit discovery sector, together with industry, can have an active role in these ambitions.

It is currently estimated that all of the FDA-approved drugs collectively address just 324 targets (of which 266 are human) [39]. Hit discovery, therefore, is ripe for innovation to produce the screening platforms that can address the large repertoire of undrugged targets. Cell-based screening has emerged as a more physiological alternative to isolated target-based approaches, with the advantage of not presuming which target on a particular pathway will be best to target [40], yet its full adoption has been hindered by robustness issues, difficulties in target deconvolution [41] and the undercurrent of reluctance among medicinal chemists to deal with the potential for complex SARs in the emergent chemical matter. Hit discovery in this sector should have the confidence and freedom to tackle pathway-based assays that provide a repertoire of potential targets and intervention points for chemical matter. This approach can contribute markedly to identifying the novel druggable mechanisms in disease-relevant pathways. In cases in which the pathway is well defined, the target is overexpressed and the required deconvolved steps are available, target identification and confirmation of cell-based screening hits can be straightforward: however, further advances are required to find a systematic, broadly applicable approach for hits with no *a priori* knowledge of the target.

There are numerous targets and mechanisms that have already established good validation *in vitro* but require progression to *in vivo* disease models to further assess their therapeutic potential. This is often hindered by the inability to identify or develop drug-like chemical matter. Our ability to address these difficult targets (e.g. protein–protein interactions and protein–RNA interactions) can be improved by focussing on the development of suitable alternative assay methodologies. For example, there is increasing confidence that fragment-based approaches offer improved opportunities for screening against difficult targets, such as protein–protein interactions, that standard biochemical HTS cannot offer [42]. The continued development of biophysical technologies, such as SPR, that can support measurement of low-affinity ligands, perhaps at multiple sites on a binding partner without total dependence on X-ray crystallography, will be vital to exploit the druggability of this target class [43].

Drives to enhance the efficiency of hit triaging and progression decisions will come from developments in many disciplines, not least chemoinformatics applications. Whatever the nature of the biological information at hit stage, its information content and physiological relevance will be a major factor in the downstream success of any compound series. The availability of human primary cells for screening in primary mode is very limited, but this could be abridged through progress being made in *in vitro* human stem cell culture, differentiation and re-programming. Advances in this field are aimed at overcoming the esoteric, ill-defined growth factor or serum component relevance and genetic-modification-based protocols historically associated with differentiation protocols. Several academically based groups have been identifying small molecules that could mimic these processes (reviewed in Refs. [44,45]). Academic screening centres are now developing high-throughout screening platforms [46] as part of a more comprehensive attempt to identify small molecules to direct differentiation of stem cells towards authentic, human-drug-discovery-relevant cell models such as cardiomyocytes, hepatocytes and pancreatic β-cells. The marriage of authentic human cell systems with technologies that enable real-time analysis of multiple cellular events will result in a worthwhile contribution towards making more informed judgements in hit triaging. Advances in high-throughput microscopy and image analysis have the potential to deliver these possibilities by promoting this application from the realms of secondary screening into a new era of routine primary screening [47,48]. The advances in label-free screening technologies evident in industry in recent years [49–51] will be increasingly adopted by academic screening centres as its instrumentation base matures. As a result of the collective ability of these technologies to support screening of endogenous targets in cellular systems and reveal mode-of-action information in both cell and isolated target scenarios, these platforms will also support the march of hit discovery towards improved physiological relevance and better informed hit selection.

Innovation in academic screening and its contribution to drug discovery will also come from the evolution of its operating models. There is increasing evidence of pharmaceutical companies wanting to marry their in-house cell models with chemical matter developed in the academic sector, such as Eli Lilly's open innovation programme. A sign that a new era of collaboration might be upon us comes from early indications that companies are now considering sharing the riches of their corporate collections with academic screening centres to further exploit their chemical matter in novel target systems and/or support neglected disease drug discovery.

## Concluding remarks

There are numerous areas of drug discovery in which academically contextualized groups can provide a pre-competitive harbour for the development and testing of new targets, methods and paradigms that can be implemented one day to improve drug discovery productivity. Applied research in this sector will no doubt lead to validation of new targets and mechanisms, new lead matter, new methodologies and production of trained staff. There are sceptics who suggest that the level of investment in the NIH initiative does not equate to tangible outputs [52], but it is crucial to allow a new sector such as this time to establish itself, diversify its approaches and grow towards a crucial mass of activity. It is also strategically important to ensure that as the academic drug discovery sector develops, it remains operationally and culturally distinctive from the pharmaceutical sector so that they can work in synergy rather than in competition. As the pharmaceutical industry re-organizes itself to respond to highly challenging times, the academic sector is in a position to help underpin drug discovery research. In doing so, it will no doubt emerge over the next decade as an ever more credible and worthwhile partner for industry in the future discovery of new medicines. The increasing potential of this industry–academic liaison has been reviewed recently [53].

For universities considering hosting a screening centre, the primary advice is to align these operations with the academic strengths of the organization in question and to have chemistry embedded to enable the progression of projects in a meaningful and timely manner. Besides having an important part to play in target validation, screening centres have the potential and opportunity to move away from the individual molecular-target approach and embark upon novel target discovery through strategically coupled phenotypic screening and target identification platforms. This represents a clear opportunity for the sector to distinguish itself from industry-led approaches and, ultimately, to provide important new knowledge to feed into both the scientific literature and future pharmaceutical industry pipelines.

## Figures and Tables

**Table 1:** Examples of some of the disciplines, technologies and automation instrumentation listed on webpages as available at one or more of 26 academic screening facilities reviewed online

**Disciplines**	**Automated liquid handling instruments**	**Detection technologies**	**Plate readers**
Atomic absorption spectroscopy	Aquarius (Tecan)	AlphaScreen	Analyst (Molecular Devices)
Automated microscopy	Biomek 3000 (Beckman)	Calcium flux	Acquest (Molecular Devices)
Computational chemistry	Biomek NX (Beckman)	Cellular imaging	ArrayScan/ArrayScan VTI (Thermo)
Drug metabolism and pharmacokinetics	Biomek FX (Beckman)	Dynamic mass redistribution	Bioimager Pathway 435 and 855 (Becton Dickinson)
Flow cytometry	Bravo (Velocity 11)	Fluorescence intensity	Epic (Corning)
High-content screening	CyBiWell (CyBio)	Fluorescence polarization	EnVision/EnVision Xcite (PerkinElmer)
High-throughput screening	Evolution P3 (Tecan)	Homogeneous time-resolved fluorescence (HTRF)	Evolution (Tecan)
Medium-throughput screening	FlexDrop (PerkinElmer)	Luminometry	FlexStation 3 (Molecular Devices)
Medicinal chemistry	Freedom EVO (Tecan)	Microfluidic separation-based assays	FLUOstar OPTIMA (BMG)
NMR screening	Hummingbird (Digilab Genomic Solutions)	Patch-clamp	Genios Pro (Tecan)
Phenotypic screening	JANUS (PerkinElmer)	Radiometry	ImageXpress Micro (Molecular Devices)
siRNA libraries	Multidrop (Thermo)		IN Cell 1000 (GE Healthcare)
Small-molecule libraries	MultiPROBE II Plus (PerkinElmer)		LabChip 3000 (Caliper)
Structural biology	Pin tools (V & P Scientific)		MicroBeta TriLux (PerkinElmer)
	PlateMate 2x2 (Thermo)		NEPHELOstar (BMG)
	PlateMate Plus (Thermo)		NOVOstar (BMG)
	Precision 2000 (BioTek)		PatchXpress (Molecular Devices)
	TekBench (Hamilton)		PHERAstar (BMG)
	Vprep (Velocity 11)		POLARstar (BMG)
	WellMate (Thermo)		Safire/Safire 2 (Tecan)
			SpectraMax/M5 (Molecular Devices)
			TopCount (PerkinElmer)
			Victor 2/Victor 3 (PerkinElmer)

## Box 1 A selection of some representative ‘academic’ screening facilities

**Table:**

**Drug Discovery Centre, London, UK**
http://www1.imperial.ac.uk/medicine/research/institutes/drugdiscoverycentre/
Remit: Translational biology for ICL discoveries.
Focus: Currently, cancer, anti-infectives, Alzheimer's, inflammation, anaesthesia and transplantation.
**Medical Research Council Technology – Centre for Therapeutics Discovery, London, UK**
http://www.mrctechnology.org/CO_DD.htm
Remit: To translate MRC-funded scientific discoveries into commercial products for healthcare benefit and to re-invest in future research.
Focus: Wide target range across broad therapeutic areas.
**University of Dundee – Drug Discovery Unit, Dundee, UK**
http://www.drugdiscovery.dundee.ac.uk/
Remit: Translational drug discovery for unmet medical needs and neglected diseases.
Focus: Tropical diseases, novel targets and mechanisms, and stem cells.
**Cancer Research Technology Discovery Laboratories, Cambridge and London, UK**
http://www.cancertechnology.com/pages/about_devlab_drugdisc.html
Remit: To translate CRUK-funded research into novel cancer therapies.
Focus: Broad target approach to drug discovery and biotherapeutic development in cancer.
**Cancer Research UK Centre for Cancer Therapeutics, London, UK**
http://www.icr.ac.uk/research/research_sections/cancer_therapeutics/index.shtml
Remit: To identify novel genes and pathways as targets and to implement novel technologies.
Focus: HTS and high-content screening (HCS) including phenotypic screening, and target families including kinases, ATPases, protein–protein interactions and signalling pathways.
**Drug Discovery Portal, Glasgow, UK**
http://www.ddp.strath.ac.uk/index.php
Remit: Partnership drug discovery.
Focus: Virtual screening and structure- and ligand-based design.
**MRC Centre for Developmental and Biomedical Genetics, Sheffield, UK**
http://cdbg.shef.ac.uk/
Remit: Generation of animal models of human disease using a translational approach.
Focus: Developmental studies in zebrafish and drosophila.
**European Molecular Biology Laboratory, Heidelberg, Germany**
http://www.embl.de/services/core_facilities/chemcore/index.html
Remit: To open up screening opportunities to research groups within and outside EMBL.
Focus: In the Chemical Biology Core Facility, to support academic partners in primary and secondary assay development, screening and chemical optimization.
**European ScreeningPort, Hamburg, Germany**
http://www.screeningport.com/
Remit: To offer fee-for-service screening to academic institutions.
Focus: Diverse target classes and therapeutic areas welcomed; utilize industry-standard platform technologies.
**Leibniz-Institut für Molekulare Pharmakologie, Berlin, Germany**
http://www.fmp-berlin.de/index_e.html
Remit: Open-access platform for academic projects.
Focus: Fragment screening by NMR; ChemBioNet compound collection repository.
**École Polytechnique Fédérale de Lausanne – Biomolecular Screening Facility, Basel, Switzerland**
http://bsf.epfl.ch/
Remit: To provide access to EPFL researchers and affiliates to molecular screening infrastructure, expertises and collections of molecules.
Focus: Cancer, neurobiology and infectious diseases.
**University of Leuven – Centre for Drug Design and Discovery (CD3), Leuven, Belgium**
http://lrd.kuleuven.be/en/entrepreneur/collaboration/cd3/about.htm
Remit: To collaborate with academic research groups and small companies on innovative targets or approaches.
Focus: Viral, bacterial and fungal diseases; central nervous system (CNS) disorders and cancer.
**Broad Institute, Cambridge, MA, USA**
http://www.broad.mit.edu/
Remit: To capitalize on genome-related science and discoveries.
Focus: Chemical Biology Platform encompassing chemistry, informatics and screening using HTS, HCS, phenotypic screens and RNAi screening.
**NIH Chemical Genomics Center, Rockville, MD, USA**
http://www.ncgc.nih.gov/
Remit: To provide a national resource in chemical probe development.
Focus: To develop chemical probe research tools relevant to physiology and disease; includes online publicly available NCGC Assay Guidance Manual.
**Johns Hopkins University – ChemCORE Facility, Baltimore, MD, USA**
http://www.hopkinschemcore.org/
Remit: To provide comprehensive compound screen services and technology development.
Focus: Special expertise with ion channels, transporters and G-protein-coupled receptors.
**The Scripps Research Institute – Translational Research Institute, Florida, USA**
http://www.scripps.edu/florida/tri/
Remit: Advanced Technologies and Drug Discovery components to the Translational Research Institute.
Focus: Broad-based discovery structure including HTS, proteomics, flow cytometry, NMR, medicinal chemistry and informatics.
**Harvard Medical School Screening Facility (ICCB Longwood), Boston, MA, USA**
http://iccb.med.harvard.edu/
Remit: To provide a staff-assisted screening model to internal and external visiting researchers.
Focus: Small molecule, RNAi and siRNA screening services.
**Memorial Sloan-Kettering Cancer Center – High-Throughput Screening Core Facility, New York, NY, USA**
http://www.mskcc.org/mskcc/html/52147.cfm
Remit: To provide cost-effective high-throughput screening facilities to local investigators.
Focus: Primarily cancer, although all projects considered.
**St Jude Children's Research Hospital – Chemical Biology and Therapeutics Department HTS Core Facility, Memphis, TN, USA**
http://www.stjude.org/basicscience
Remit: To discover and develop novel chemical entities that increase understanding of the pathophysiology of or function as therapeutic leads for the treatment of catastrophic paediatric illnesses.
Focus: Primarily childhood cancers.
**University of Kansas – High-Throughput Screening Laboratory, Lawrence, KS, USA**
http://www.hts.ku.edu/index.php
Remit: To provide screening capabilities both locally and nationally.
Focus: Small-molecule library screening; siRNA screening.
**Southern Research Institute, Birmingham, AL, USA**
http://www.southernresearch.org
Remit: Drug Discovery Research Team focussing on cancer, infectious disease and neuroscience.
Focus: HTS using BSL3 pathogens; antiviral, antimicrobial and anticancer screening.
**University of British Columbia – Centre for Drug Research and Development, Vancouver, Canada**
http://www.cdrd.ca/facilities-expertise/drug-screening
Remit: To advance promising discoveries to a commercially attractive stage and to build a collaborative research infrastructure.
Focus: Cancer, CNS, diabetes and infectious diseases.
**McGill University Life Sciences Research Complex, Montreal, Canada**
http://www.lifesciencescomplex.mcgill.ca/hts-hcs
Remit: To facilitate the development of chemical biology programmes in academic laboratories using a staff-assisted screening model.
Focus: HTS and HCS projects.
**Walter and Eliza Hall Institute of Medical Research – WEHI Biotechnology Centre HTCS Facility, Melbourne, Australia**
http://www.biotechcentre.net.au/HTCS/htcs.html
Remit: Open-access screening facility.
Focus: Molecular-target and cell-based screening approach using conventional technology platforms, including surface plasma resonance technology.
**Eskitis Institute for Cell and Molecular Therapies, Griffith University, Brisbane, Australia**
http://www.griffith.edu.au/science/eskitis-institute-cell-molecular-therapies
Remit: To investigate novel drug- and cell-based therapies for cancer, infection, neglected diseases, neurodegenerative diseases and stem cell biology.
Focus: The ‘NatureBank’ natural product resource, adult stem cell biology, breast cancer and Parkinson's disease.
**Institut Pasteur Korea, Kwanggyo, South Korea**
http://www.ip-korea.org/
Remit: To further the Institut Pasteur's mission by applying Korean technology and competence in areas of neglected diseases.
Focus: Infectious and chronic diseases, nanotechnology, PhenomicScreen™ and high-throughput flow cytometry.
